# Knowledge, attitudes, and practices towards fetal alcohol spectrum disorder in the New Zealand social and community sector: An online survey

**DOI:** 10.1177/17446295231172234

**Published:** 2023-04-24

**Authors:** Jessica C. McCormack, Joanna Ting Wai Chu, Holly Wilson, Juma Rahman, Samantha Marsh, Chris Bullen

**Affiliations:** Food Science, 2495 University of Otago, Dunedin, New Zealand; National Institute for Health Innovation, School of Population Health, 1415 The University of Auckland, Auckland, New Zealand; National Institute for Health Innovation, School of Population Health, 1415 The University of Auckland, Auckland, New Zealand; National Institute for Health Innovation, School of Population Health, 1415 The University of Auckland, Auckland, New Zealand; National Institute for Health Innovation, School of Population Health, 1415 The University of Auckland, Auckland, New Zealand; National Institute for Health Innovation, School of Population Health, 1415 The University of Auckland, Auckland, New Zealand; National Institute for Health Innovation, School of Population Health, 1415 The University of Auckland, Auckland, New Zealand

**Keywords:** fetal alcohol, social and community workers, knowledge, attitudes, practices

## Abstract

**Background:** Fetal Alcohol Spectrum Disorder (FASD) is a common neurodevelopmental disorder but may be underrecognized and misunderstood by people who provide health and social support services. The aim of the research is to understand the FASD knowledge, attitudes, and practices among people employed by the social and community sector in New Zealand. **Methods:** We conducted an online survey of people working in the New Zealand social and community sector (i.e., social workers, support workers). The survey focused on the following areas: awareness of FASD; knowledge and beliefs about FASD; the impact of FASD on professional practice; and training needs. **Results:** Most participants reported a basic understanding of FASD, however only 5% felt very well prepared to support someone with FASD. A large majority of participants believed that FASD diagnosis may be stigmatising for individuals or families. **Conclusion:** There is a need to improve training, professional development, and workplace support for social and community workers in New Zealand to support people with FASD.

## Introduction

Fetal Alcohol Spectrum Disorder (FASD) is a diagnostic term that describes the neurological and physical effects of prenatal exposure to alcohol (Cook et al., 2016) and is one of the most common forms of neurodevelopmental disorder. The global prevalence of FASD is estimated at 7.7 per 1000 births, but estimates vary considerably across countries (Lange et al., 2017). In New Zealand, the Ministry of Health estimates that of every 100 live births, 3 to 5 are affected by alcohol (Ministry of Health, 2022), although this is likely to be an underestimate of the true number of those living with FASD due to difficulties accessing a formal diagnosis (Coons et al., 2018; Mukherjee et al., 2013).

People living with FASD face challenges in their daily lives due to impairments across multiple neurocognitive domains including memory, cognition, language, executive function, social skills and attention (Cook et al., 2016). They often require support from professionals in many sectors including education, health, and social and community services (Petrenko et al., 2014). Without adequate support, individuals with FASD may experience difficulties that can lead to school disruption, poor mental health, substance misuse, victimization, engagement with the justice system, and incarceration (McLachlan et al., 2020; Streissguth et al., 2004). In New Zealand, FASD is not recognised as a disability, therefore people living with FASD can only access disability support services if they have been diagnosed with a physical or intellectual disability, requiring an IQ less than 70 (Disability Rights Commissioner and Children’s Commissioner, 2021). To be diagnosed with FASD people do not need to have an intellectual disability and often do not meet the government criteria**,** restricting the government funded support that people with FASD can access in New Zealand.

The social and community sector, which includes social workers, community support workers (i.e., support workers employed in home and community settings to help people live independently), mental health workers, and youth workers, plays a major role in supporting people with FASD in the community. People living with FASD may be referred to community services due to secondary or tertiary impacts associated with FASD such as mental health issues, addiction, homelessness, and under or unemployment (Moore and Riley, 2015). Lack of knowledge or awareness of FASD by these support professionals is a major barrier for families when attempting to access services or support for FASD (Coons et al., 2018; Petrenko et al., 2014). For example, families report challenges in making themselves understood by providers when accessing services in the community (Mukherjee et al., 2013). This lack of knowledge can contribute to misdiagnosis, misinterpretation of behaviours, inadequate service provision, and failure to intervene to prevent adverse life outcomes (Petrenko et al., 2014).

While a number of previous studies have evaluated the knowledge, attitudes, and practices (KAP) of healthcare and justice professionals in relation to FASD and prenatal alcohol exposure (McCormack et al., 2022), only four studies have included professionals from the social and community sector. A small survey in 1997 of US social workers (N=44) found almost two-thirds had received training related to Fetal Alcohol Syndrome (a diagnosis within FASD), and those who had received training scored higher in their knowledge of Fetal Alcohol Syndrome in a true-false test (Wolff, 1998). In contrast, a 2009 US nation-wide survey of professionals working in the social services (N=1902) found that social services professionals were knowledgeable when it came to prevention of FASD and alcohol-exposed pregnancies, but were less knowledgeable about its epidemiology, diagnosis, and intervention strategies (Johnson et al., 2010). More than 70% reported that they felt under-prepared to manage or co-ordinate care for a child with FASD. A 2014 UK survey of health professionals (N=505) that included social workers (n=79) found that while almost all respondents were aware of FASD, less than a third believed they had sufficient information to advise patients about safe alcohol consumption (Mukherjee et al., 2015). More recently, a 2020 qualitative study of UK social workers working with children with FASD (N=8) found that a lack of knowledge of FASD was linked to difficulties managing children suspected of FASD (Gilbert et al., 2021).

In New Zealand, there has been very limited research on social and community sector workers in relation to FASD. We aimed to measure the degree to which this important group is aware of and has the knowledge and attitudes needed to support individuals with FASD and their families, with a view to informing training resources and guidelines.

## Methods

### Design

We conducted an online KAP survey of people working in the social and community sector in New Zealand. The survey consisted of multi-choice, true-false, and open-ended questions and took 5-10 minutes to complete. The survey was adapted from a survey conducted of New Zealand educators (Chu et al., 2022), which was modelled on previous surveys of health and justice professionals (Payne et al., 2011; Cox et al., 2008; Wouldes, 2009; Mutch et al., 2013). The development of that survey is described in full in Chu et al. (2022). We adapted the survey questions in Chu et al. (2022) to describe the work of people in the social and community sector and sought feedback from sector workers to ensure that the wording was appropriate. Based on findings from our previous survey with educators, we added questions regarding the potential stigma associated with FASD and whether participants believed individuals with FASD are entitled to receive state-funded support. The survey covered four domains: awareness of FASD, knowledge and beliefs about FASD, impact of FASD on professional practice and experience with FASD, and training and information needs relating to FASD. The survey also collected demographic information, location (i.e., rural, urban, or city centre), role in the sector, types of work setting, and years working in the sector.

### Study Population

Participants were eligible to participate in the survey if they were aged ≥18 years, resident in New Zealand, able to read and speak English, currently employed in New Zealand, and have contact with clients in their work. Based on our previous survey, we aimed to recruit 300 participants across the different groups working in the social and community sector (i.e., social workers, community support workers, youth workers, and mental health workers).

### Recruitment

We recruited participants over five weeks from 14 February 2022 to 21 March 2022 through mailing lists, social media platforms (Facebook, Twitter) and e-newsletters to professional groups. Social medial links were shared through provider and advocacy networks including the New Zealand FASD Care Action Network (FASD-CAN) and Australia New Zealand FASD Clinical Network (ANZFASD-CN). We also sent email invitations to senior staff in social services organisations, such as Oranga Tamariki (the New Zealand Ministry responsible for childrens’ wellbeing), and to social and community services providers, inviting their staff to participate in the survey. On completion of the survey, in recognition of their time completing the survey, participants were invited to enter a prize draw to receive 1 of 10 $NZ50 vouchers.

All participants were informed of the aims and focus of the survey before via an online participant information sheet before starting the survey; completion of the survey was equivalent to consent for their answers to be used in their research.

### Analysis

All data were collected and managed via an online survey created in a REDCap database (Harris et al., 2019) hosted by the University of Auckland and exported to SAS version 9.4 (SAS Institute Inc., Cary, NC, USA, 2016) for analysis. Continuous variables were summarised as frequencies, means and standard deviations (SD), medians and interquartile range. Categorical variables were summarised as percentages, with missing data excluded. Comparisons were made using Chi-square tests and Wald tests to assess statistical significance. The significance level was set at 5% with two-tailed tests.

Open-ended answers were exported to NVivo (QSR International Pty Ltd, 2020) for analysis by the first author (JCM) using reflexive thematic analysis methods (Braun and Clarke, 2022) The first phase of analysis was familiarisation, followed by coding of open-ended responses using semantic codes (Terry and Hayfield, 2020). Once all data had been coded, we built thematic maps to identify clusters around central themes with shared meaning, which were then reviewed by JCM and JC.

## Results

In total, 225 people responded to the online survey invitation. We excluded 38 responses that were from people not employed in the social sector, were only involved with administration, or who did not complete the screening question. Our final sample consisted of 187 participants from the New Zealand social and community sector.

The largest group of professionals in our sample was social workers (n=71, 38.0%), followed by Other (n=34, 18.1%), which included students, probation and corrections officers, health promotors, and advisors. The remaining participants were mental health workers (n=23, 12.3%), community support workers (n=27, 14.4%), youth workers (n=12, 6.4%), case managers (n=9, 4.8%), and clinical workers (n=9, 4.8%), a category that included psychologists, therapists, counsellors, and nurses (Table 1). Three participants did not select a role within the social and community sector.

**Table 1.** Baseline characteristics of participants by professional group: count (%).

	Social worker	Mental health worker	Community Support Worker	Case Manager	Youth Worker	Clinical Worker	Other
*N*	71	23	27	9	12	9	34
Age M (SD)	44 (11.3)	44 (14.7)	41.3 (13.6)	47 (14.5)	41.7 (12.5)	49.9 (9.1)	49.8 (13.6)
Gender							
Male	8 (11.9)	1 (4.5)	2 (7.7)	2 (22.2)	5 (41.7)	1 (12.5)	2 (6.5)
Female	58 (86.6)	21 (95.5)	24 (92.3)	7 (77.8)	7 (58.3)	7 (87.5)	29 (93.5)
Gender Diverse	1 (1.5)	0 (0)	0 (0)	0 (0)	0 (0)	0 (0)	0 (0)
Prioritised ethnicity							
Māori	13 (18.3)	4 (17.4)	3 (11.1)	6 (66.7)	4 (33.3)	1 (11.1)	4 (11.8)
Pacific	1 (1.4)	0 (0)	2 (7.4)	0 (0)	0 (0)	1 (11.1)	1 (2.9)
European	54 (76.1)	18 (78.3)	18 (66.7)	3 (33.3)	6 (50)	7 (77.8)	24 (70.6)
Asian	2 (2.8)	0 (0)	3 (11.1)	0 (0)	2 (16.7)	0 (0)	4 (11.8)
Other	1 (1.4)	1 (4.3)	1 (3.7)	0 (0)	0 (0)	0 (0)	1 (2.9)
Location							
Inner City	20 (28.2)	2 (9.1)	2 (8)	4 (44.4)	1 (7.7)	2 (22.2)	2 (5.9)
Suburbs	41 (57.7)	14 (63.6)	20 (80)	4 (44.4)	11 (84.6)	5 (55.6)	24 (70.6)
Rural	10 (14.1)	6 (27.3)	3 (12)	1 (11.1)	1 (7.7)	2 (22.2)	8 (23.5)
Years in sector							
Less than 1 year	1 (1.4)	1 (4.5)	2 (7.7)	1 (11.1)	0 (0)	0 (0)	4 (11.8)
1–2 years	12 (16.9)	3 (13.6)	8 (30.8)	1 (11.1)	1 (7.7)	1 (11.1)	2 (5.9)
3–4 years	6 (8.5)	6 (27.3)	6 (23.1)	0 (0)	1 (7.7)	0 (0)	4 (11.8)
5–10 years	23 (32.4)	2 (9.1)	6 (23.1)	1 (11.1)	7 (53.8)	3 (33.3)	9 (26.5)
11 or more years	29 (40.8)	10 (45.5)	4 (15.4)	6 (66.7)	4 (30.8)	5 (55.6)	15 (44.1)
Aware of FASD							
Yes	69 (97.2)	22 (100)	22 (84.6)	9 (100)	12 (92.3)	9 (100)	33 (91.7)
No	0 (0)	0 (0)	2 (7.7)	0 (0)	0 (0)	0 (0)	1 (2.8)

Most participants identified as female (82.9%) and identified as New Zealand European (71.1%). Additionally, 18.2% identified as Māori, 2.7% as Pacific Islander, and 8.0% as Asian or another ethnic group. Most participants were from suburban areas and most participants (67.5%) had worked in the sector for 5 or more years, with 39% reporting working in the social sector for 11 or more years. Participants worked in the following settings (multiple settings could be selected): Healthcare settings (61), Youth services (46), School or education settings (29), Addiction services (24), Prison or probation settings (13), Māori services (19), Pacific services (8), and Other (75).

## Awareness of FASD

Almost all participants (94.5%), regardless of professional group, reported that they were aware of FASD prior to the study. The most common sources of information about FASD were professional training and information (62.0%), followed by education sessions such as a conference or workshop (46.5%), colleagues (44.4%), and their own research (37.4%). Other sources of information reported by participants included professional experience with clients living with FASD or personal experience with whānau (extended family) living with FASD. Only one participant reported that they were aware of FASD through formal education.

## Knowledge of FASD

When asked to rate their knowledge or awareness of FASD, the majority of participants rated their knowledge level as a basic understanding of FASD and its effects for people with FASD (Table 2). Self-rated knowledge of FASD was lowest for case managers while mental health workers and clinicians were more likely to rate themselves as having a good understanding of FASD.

**Table 2.** Percentage and number of participants self-reported knowledge and awareness of the effects of FASD by social and community group.

	Aware of FASD, but don’t know what effects it has		Basic understanding FASD and its effects		Good understanding of FASD and its effects		Good understanding of FASD and its effects relating to social services role	
Social and Community worker group	*n*	%	*n*	%	*n*	%	*n*	%
All participants	8	4.3	97	51.9	44	23.5	31	16.6
Social Workers	0	0	41	57.7	18	25.4	12	16.9
Mental Health Workers	2	8.7	9	39.1	7	30.4	4	17.4
Community support workers	3	11.1	13	48.1	5	18.5	2	7.4
Youth Workers	1	8.3	5	41.7	3	25.0	2	16.7
Case Managers	0	0	7	77.8	2	22.2	0	0
Clinical workers	0	0	5	55.6	1	11.1	3	3.33
Other	2	3.1	15	45.5	7	21.2	7	21.2

Participants were given a list of characteristics and asked to identify which three features required for a diagnosis of FASD (central nervous system abnormality, neurological impairments, and exposure to alcohol during pregnancy). Most participants were able to identify at least two features (60.4%), but only a quarter (26.7%) identified all three. Neurological impairments and exposure to alcohol during pregnancy were identified by 89.3% and 93.1% of participants respectively, but only 30.5% identified central nervous system abnormality or dysfunction as a feature of FASD. More than a third of participants identified distinctive facial features as a feature of FASD (41.2%).

Participants were asked to estimate how common they believed FASD to be in New Zealand. The Ministry of Health estimate of FASD in New Zealand has recently been updated from 1-3% to 3-5% (Ministry of Health, 2022), therefore we treated both 1 in 50 (2%) and 1 in 20 (5%) as correct responses. Only 31.0% correctly estimated the prevalence of FASD in New Zealand (14.4% 1 in 20 and 16.6% 1 in 50) with 51.3% of participants underestimating it, 9.1% estimating that FASD affected only 1 in 10,000 people (0.01%). Underestimation of FASD was highest among youth workers (58.3%) and lowest in the Other category of worker (36.4%).

Participants were also asked to estimate how common FASD is likely to be in people involved in the justice system, including incarcerated adults, children in the care of youth justice, arrestees, and victims of crime. The prevalence of FASD in justice system is unknown in New Zealand, however, estimations from international studies indicate the prevalence to range from 10 to 36% (Hughes et al., 2016; Bower et al., 2018; McLachlan et al., 2019). Almost half of participants (49.7%) estimated that 1 in 5 people involved in the justice system is affected by FASD and 13.9% estimated that 1 in 100 or fewer people involved in the justice system were affected by FASD.

## Attitudes and Beliefs Towards FASD

**Figure 1.**
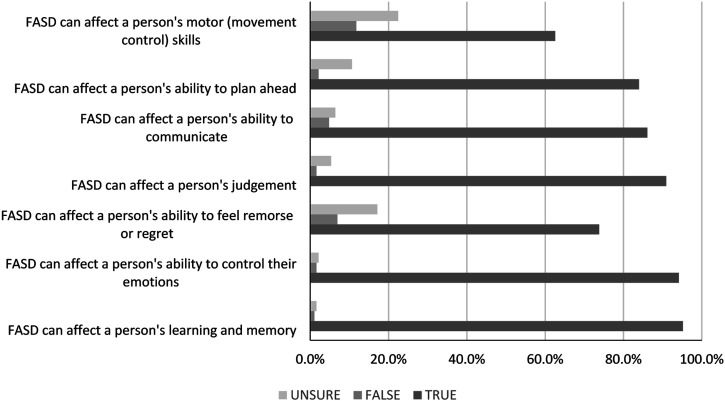
Percentage of participants endorsing the domain as affected by FASD.

A large majority of participants correctly identified the following as domains affected by FASD: learning and memory (95.2%), emotional regulation (94.1%), communication (86.1%), and forward planning (84.0%) (Figure 1). Only 56.7% correctly identified motor skills or movement control as an area affected by FASD. More than 80% of participants believed that FASD can affect a person’s judgement and two-thirds believed that FASD can affect a person’s ability to feel remorse or regret for their words or actions.

General beliefs about FASD were assessed through a series of true-false statements. A large majority correctly rejected the statements that people can grow out of FASD (85.0%), that FASD is only relevant to people under 18 years (86.5%), and that a diagnosis of FASD would NOT improve outcomes for those affected by FASD (72.6%). More than two-thirds of participants recognised that people with FASD have permanent brain damage. In response to the statement “most birth mothers who drink when pregnant know it can harm the baby” only around half of participants correctly rejected the statement (47.6%), while 28.8% reported that the statement was true. Almost half of participants believed, incorrectly, that diagnosis of FASD would enable an individual to access Disability Support Services (47.1%) and 13.0% were unsure whether this statement was true or false. Almost two-thirds of social and community sector employees believed that diagnosis of FASD might lead to a child or their family being stigmatized (64.9%).

### Experience and Practices Related to FASD

When asked if FASD was relevant to their work in the social sector, almost three-quarters of participants reported that FASD was relevant or highly relevant (74.3%), with 45.5% reporting that FASD was highly relevant. Only 13.4% reported that FASD was highly irrelevant to their work. Compared to the other groups, a greater proportion of social workers and case manager reported that FASD was irrelevant to their work in the social sector (18.3% of social workers and 33.3% of case managers), however more than half of social workers also reported that FASD was highly relevant to their work (50.7%). Youth workers most strongly endorsed FASD as relevant to their work, with two-thirds of youth workers reporting that FASD was highly relevant. When asked to report the percentage of their current clients they believed may have FASD, 66.3% reported that 10% or more of their current client may have FASD, with 10.7% reporting 60% or more.

More than 70% of social sector employees reported that they would change their practices or behaviours if someone in their care was known to have FASD. When asked to explain their answer (n=166), participants recognised that individuals with FASD have different needs and require different approaches, with many participants highlighting the need for different communication strategies and the need for support that accounts for the individuals needs and limitations and deficits. Participants also highlighted the need for different kinds of support, such as advocating on behalf of the client, educating the individual’s community and support for the family. Knowing that a client had an FASD diagnosis was also seen as giving insight or context to the individual’s behaviour: *“To know someone is to have the whole picture not half”* (Case manager). Because people with FASD have different needs, some participants noted the need for specialised support or services, or to seek out training and information to support people with FASD, with some participants reporting that their service might not be appropriate for people with FASD. One participant, however, described the need for greater vigilance towards individuals living with FASD because they are a *“danger to those around them”* (Social Worker). For those who would not change their practices or behaviour (n=25), the main reasons were because their practices were already responsive to individual differences or avoiding discriminatory behaviour towards FASD.

More than two-thirds of participants reported having a client diagnosed or undiagnosed but suspected of FASD under their care (66.8%); 11.2% reported than they had personally suspected a client of having FASD and only 15.5% reported that they had not had a client in their care with FASD. When asked about what care was provided, how they managed their care, and what information they were provided with in order to provide support, participants responded that there had been little or no support provided (n=27). Many participants reported that support was provided on the basis of a different diagnosis – such as global developmental delay, intellectual disability, or autism spectrum disorder – rather than an FASD diagnosis, with some participants noting that individuals with FASD are unable to access disability support. Similarly, many participants reported that they had received none or minimal information to help them support the client (n=54), although others reported they had received assessment reports or clinical notes (n=14) or were provided with training (n=24). In the absence of support or information, many participants had taken it upon themselves to find out more about FASD, conducting their own research online or seeking out training resources such as those provided by FASD support groups or the Oranga Tamariki Practice Centre.

Only 5.3% of participants reported that they felt very prepared to support someone with FASD if they were assigned to their care. Overall, 40.6% reported that they felt moderately prepared, while 21.9% reported that they felt somewhat unprepared and 10.2% reported that they felt not at all prepared. Just over half of case managers felt prepared to support someone with FASD (55.6%) while only 33% of clinicians felt prepared.

### Education and Training Related to FASD

One third of participants reported that they had received training relating to FASD in the last five-years (35.8%). Almost half of social workers reported receiving FASD training in the last five-years (47.9%). The type of training and duration varied across responses (n=64), from self-directed online reading or webinars to professional training and education over several weeks or months. Participants also reported a variety of different providers including Ministry of Health or District Health Board training (n=8), FASD clinicians (n=8), Caregiver Support groups (n=5), Oranga Tamariki (n=5), and overseas providers from Australia and Canada (n=5). A small proportion reported that they received training related to other neurological or neurodevelopmental conditions but not FASD (14.4%), which was most commonly reported to be training on autism spectrum disorders. Thirty-seven percent reported that they had not received training on FASD or other neurodevelopmental conditions in the last five years.

Participants were also asked if they were aware of the FASD resources and online training provided by Te Pou – a Ministry of Health funded workforce development provider. Most participants reported that they were not aware of the resources (53.5%) and less than 20% reported that they had read the resources or taken part in the online training provided by Te Pou (17.6%).

When asked what information would help prepare them (n=148) some participants highlighted the need for more training in general, especially ongoing training that continued to update people on the best practices and latest information about FASD and quick reference resources. Participants also emphasised the need for resources to be based in a New Zealand context and have meaningful engagement with Māori as *tangata whenua* (Indigenous people of New Zealand). Specific information to include in training included understanding FASD and its impact on the individual, strategies for engaging with people with FASD, and managing behaviour. A key theme from the participants was the need for information around support pathways including how to access funding for a diagnosis, what support and funding is available, what services are appropriate and where to direct families, and the need for targeted support services for FASD. Suggested sources for this information included expert advisors on FASD and peer support networks. Another important theme was the need for information specific to the client such is, detailed medical history, information from previous services, assessments and recommendations, and behavioural plans, along with co-ordination across services. The need for greater access to funding, such as Disability Support Services, for FASD and recognition of FASD as a disability was also highlighted by some participants.

The main resources requested by participants were training workshops (75.9%), online resources (73.8%), and FASD-informed strategies (72.7%) and protocols to support people with FASD (72.2%). All resources were endorsed as being helpful to their work by 60% or more of participants. The only resources with relatively low endorsement were FASD diagnostic checklists, but this was still endorsed by most participants (62.0%). Other resources requested by participants (n=11) included interventions built upon Kaupapa Māori and Pasifika values to delivered interventions tailored to the needs of Māori and Pacific communities, professional forums, and hui (meeting) opportunities, and adequate funding for assessment and intervention of FASD.

When asked about potential challenges for working with individuals living with FASD, three main themes emerged. The first was around having the appropriate knowledge and skills for working with people with FASD – both personally and in other staff members: *“My team do not know much about FASD, let alone how to work with people who have FASD. This is a huge challenge”* (Clinical worker). Participants noted several barriers to ensuring that those working with FASD had the appropriate level of skill such as lack of time and opportunities for training, having incomplete information about clients, and lack of support from management or other professionals to address FASD. Another theme was around the lack of funding or services to support people with FASD. Even where support was provided participants noted that the support was insufficient or inappropriate for FASD, especially where support was expected to be completed in a short period of time or to address a specific problem, rather than long-term support to address the daily challenges of living with FASD: *“Short term intervention is not suited for those with FASD and their families”* (Social Worker). This theme also included the inaccessibility of assessment services due to limited diagnostic services, especially outside of major cities, and funding. The final theme was the stigma attached to FASD: *“Being labelled as ‘bad’ rather than struggling with FASD”* (Social Worker). Stigma was seen as a barrier to diagnosis and accessing appropriate supports. For example, *“denial due to the embarrassment or guilt where the birth mothers actions have effected the child”* (Community Worker) might make it hard for families to accept the diagnosis or make families reluctant to seek help for fear of judgment from others. Lack of awareness of FASD in the broader community contributed to stigma as well as outdated knowledge and myths about FASD.

## Discussion

This research is the first in New Zealand to survey workers in the social and community sector about their knowledge, attitudes and practices in relation to FASD. We found that, while social and community workers had some knowledge of FASD, gaps in knowledge and inaccurate beliefs are common, which need to be addressed so social and community workers can provide best possible support for people living with FASD. Social and community workers self-reported a basic knowledge of FASD and some held beliefs that were inconsistent with correct information about FASD. This was similar to findings of a previous survey of education workers (Chu et al., 2022) and consistent with studies that show social workers have basic knowledge about FASD (Johnson et al., 2010; Gilbert et al., 2021).

The majority of participants underestimated the prevalence of FASD in New Zealand, with some participants believing FASD to be very rare (i.e., 1 in 10000). In contrast, almost half of participants believed that FASD was very common (i.e., 1 in 5) in people involved in the justice system such as incarcerated adults, children in the care of Youth Justice, arrestees, and victims of crime). In New Zealand there has been no research into prevalence of FASD in criminal justice populations (McCormack et al., 2021) or in justice involved individuals, however, empirical studies from Canada and Australia suggest that the prevalence of FASD in justice population to range from 10 to 36% (Hughes et al., 2016; Bower et al., 2018; McLachlan et al., 2019), a higher prevalence than in the general populations (Popova et al., 2019). If FASD goes undiagnosed in the justice system people face challenges in accessing fair and equitable treatment (Reid et al., 2020). The association of FASD with people involved in the justice system may be partly due to the high profile criminal case of *Pora v The Queen,* a notorious miscarriage of justice where a Māori teenager with FASD was falsely imprisoned for 22 years (Freckelton, 2016).

Nearly half of the participants incorrectly believed that distinctive facial features are a feature of FASD; in reality only 10% of people with FASD show distinctive facial features (Andrew, 2010). This is particularly problematic as often people with FASD do not display physical features that make their FASD visible, which has the potential to result in inappropriate support for FASD. Also, almost half of the social and community workers believed that a diagnosis of FASD would enable access to disability support services, however, to access disability support services in New Zealand people need to have an IQ level of 70 or less, which excludes the 80% of people with FASD who do not meet this criterion (Disability Rights Commissioner and Children’s Commissioner, 2021). Few participants correctly recognized central nervous system abnormality as a feature of diagnosis for FASD, this could be due to lack of knowledge about the central nervous system or diagnosis for FASD as social and community workers are not usually involved in diagnosis but support for people with FASD. Several participants noted that the lack of funding and support was a barrier for them to be able to support people living with FASD. As social and community workers in New Zealand potentially work closely with and support families and people living with FASD it is important that they have the correct information about diagnosis and support to guide people to the support available. Further, perhaps this lack of knowledge and the recognition of the lack of funding in New Zealand point to the need for more funding to be available to support people with FASD in New Zealand.

Most social and community workers believed that FASD diagnosis might lead to a family or child being stigmatized. Participants also raised that potential stigma towards people with FASD as a barrier to diagnosis and accessing support. Stigma can be a major barrier towards individuals obtaining an appropriate diagnosis where FASD is suspected (Mukherjee et al., 2013), especially where individuals may not qualify for Disability Support Services. Only half of participants correctly reported that most birth mothers who drink when pregnant are unaware of the potential harm to the baby, suggesting social and community workers may hold stigmatizing beliefs about women who given birth to children prenatally exposed to alcohol. Previous studies have found that negative attitudes and stereotypes about mothers of children prenatally exposed to alcohol are common in the general public (Roozen et al., 2022). Findings from our survey suggest that the belief that FASD is a stigmatizing diagnosis is pervasive, even amongst those that have a relatively good knowledge and awareness of FASD, which could prevent families from seeking diagnosis and support for FASD.

Participants in our survey endorsed all resources and some had self-sought information, which may be indicative of the scarcity of information available for workers in this sector. Comparatively fewer participants endorsed diagnostic checklists, likely because this type of material would not be relevant to most participants working in frontline social and community roles. Many reported that a lack of training, workplace support and resources to support them to support someone with FASD was a barrier to working with people with FASD, suggesting that there is limited professional resources and training about FASD and further professional development is important. Workplace training sessions about FASD with justice professionals, incorporating videos, workshop and group discussions, improved the knowledge, attitudes and provided skills to justice professionals (Passmore et al., 2021), showing the benefits of professionals training about FASD.

Whereas findings from our survey were generally in line with those from a previous survey of education workers (Chu et al., 2022), a greater portion of the social and community workers reported that they had experience working with clients with FASD. However, a larger proportion of social and community workers than educators reported that FASD was not relevant to their work. As noted by Gilbert et al., (2021), a lack of knowledge or skill was identified as a major challenge to their work in addressing FASD. Only 5% of respondents felt well prepared to support someone with FASD, consistent with studies demonstrating that 70% of people working in social services believed they were not prepared to work with someone with FASD (Johnson et al., 2010). Therefore, even though people had experience supporting people with FASD, they did not feel well prepared, perhaps due to the lack of support available in their workplaces.

The largest group of respondents was social workers, a group that typically work across multiple settings and are likely to be a key point of contact for individuals and families affected by FASD. In general, most social workers felt prepared to work with people with FASD and described themselves as having a basic understanding of FASD. While a large majority believed that FASD was relevant or highly relevant to their work, nearly 1 in 8 social workers believed that FASD is highly irrelevant to their work. Taken together, more education and support provided to social workers would enable them to provide better support to people with FASD.

### Strengths and limitations

Our survey has several strengths: it is the first in New Zealand to target the social services sector despite workers having roles that bring them into contact with people and families facing the challenges of FASD. Among the few international surveys that have targeted this group the study is one of the largest, enabling social workers attitudes to be explored in a representative sample. This surveys similar method with our recent survey of education workers (Chu et al., 2022), enables the knowledge, attitudes and practices of education workers to be compared with social and community workers in New Zealand.

Limitations include the use of an online survey to collect data, potentially biasing responses towards social and community workers with previous knowledge and experience of FASD, as they may have self-sought information about FASD, and limiting their generalisability. We did not meet our recruitment targets overall or for any group of respondents and the small number of responses for some groups limited our ability to make comparisons across or within groups. Future research in this field should involve multiple sectors to allow for direct comparisons between the sectors and would be strengthened by including in-depth qualitative interviews of their clients and families living with FASD.

## Conclusion

Social and community workers in New Zealand have a basic understanding of FASD, but there are gaps in their knowledge, around the diagnosis and support services available to people with FASD. With social and community workers likely being a key contact and support for people with FASD in New Zealand, few felt well prepared to support people with FASD. Therefore, there is a need for continued education, professional development, and workplace policies to enable the best social and community support for people living with FASD in New Zealand.

## References

[bibr1-17446295231172234] Andrew G (2010) Diagnosis of FASD: An Overview. Fetal Alcohol Spectrum Disorder. pp.127–148.

[bibr2-17446295231172234] Bower C Watkins RE Mutch RC , et al. (2018) Fetal alcohol spectrum disorder and youth justice: a prevalence study among young people sentenced to detention in Western Australia. BMJ Open 8(2): 019605. 10.1136/bmjopen-2017-019605 PMC5829911 29440216

[bibr100-17446295231172234] Braun V Clarke V (2022) Conceptual and design thinking for thematic analysis. Qualitative Psychology 9(1): 3–26.

[bibr3-17446295231172234] Chu J McCormack J Marsh S et al. (2022) Knowledge, Attitudes and Practices Towards Fetal Alcohol Spectrum Disorder in New Zealand Educators: An Online Survey. Journal of Intellectual Disabilities 0(0): 1–15. 10.1177/17446295221104618 35634949

[bibr4-17446295231172234] Cook JL Green CR Lilley CM , et al. (2016) Fetal alcohol spectrum disorder: a guideline for diagnosis across the lifespan. Canadian Medical Association Journal 188(3): 191–197. 26668194 10.1503/cmaj.141593 PMC4754181

[bibr5-17446295231172234] Coons KD Watson SL Yantzi NM , et al. (2018) Adaptation in families raising children with fetal alcohol spectrum disorder. Part II: What would help. Journal of Intellectual & Developmental Disability 43(2): 137–151.

[bibr6-17446295231172234] Cox LV Clairmont D Cox S (2008) Knowledge and attitudes of criminal justice professionals in relation to Fetal Alcohol Spectrum Disorder. Journal of Population Therapeutics and Clinical Pharmacology 15(2): e306-313. 18678920

[bibr7-17446295231172234] Disability Rights Commissioner Children’s Commissioner (2021) Fetal Alcohol Spectrum Disorder: A Call to Action.

[bibr8-17446295231172234] Freckelton I (2016) Fetal Alcohol Spectrum Disorders, Expert Evidence and the Unreliability of Admissions during Police Interviews. Psychiatry, Psychology and Law 23(2): 173–183.

[bibr9-17446295231172234] Gilbert DJ Mukherjee RA Kassam N , et al. (2021) Exploring the experiences of social workers in working with children suspected to have fetal alcohol spectrum disorders. Adoption & Fostering 45(2): 155–172.

[bibr10-17446295231172234] Harris PA Taylor R Minor BL , et al. (2019) The REDCap consortium: Building an international community of software platform partners. Journal of Biomedical Informatics 95: 103208. 31078660 10.1016/j.jbi.2019.103208 PMC7254481

[bibr11-17446295231172234] Hughes N Clasby B Chitsabesan P , et al. (2016) A systematic review of the prevalence of foetal alcohol syndrome disorders among young people in the criminal justice system. Cogent Psychology 3(1): 1214213.

[bibr12-17446295231172234] Johnson ME Robinson RV Corey S , et al. (2010) Knowledge, attitudes, and behaviors of health, education, and service professionals as related to fetal alcohol spectrum disorders. International Journal of Public Health 55(6): 627–635. 20809348 10.1007/s00038-010-0186-8

[bibr13-17446295231172234] Lange S Probst C Gmel G , et al. (2017) Global Prevalence of Fetal Alcohol Spectrum Disorder Among Children and Youth: A Systematic Review and Meta-analysis. JAMA Pediatr 171(10): 948–956. 28828483 10.1001/jamapediatrics.2017.1919 PMC5710622

[bibr14-17446295231172234] McCormack JC Chu JTW Marsh S , et al. (2022) Knowledge, attitudes, and practices of fetal alcohol spectrum disorder in health, justice, and education professionals: a systematic review. Research in Developmental Disabilities 131: 104354. 36375286 10.1016/j.ridd.2022.104354

[bibr15-17446295231172234] McCormack JC McGinn V Marsh S , et al. (2021) Fetal alcohol spectrum disorder and prisoners: the need for research-informed action. The New Zealand Medical Journal (Online) 134(1533): 118–121. 33927431

[bibr16-17446295231172234] McLachlan K Flannigan K Temple V , et al. (2020) Difficulties in Daily Living Experienced by Adolescents, Transition-Aged Youth, and Adults With Fetal Alcohol Spectrum Disorder. Alcoholism: Clinical and Experimental Research 44(8): 1609–1624. 32472600 10.1111/acer.14385

[bibr17-17446295231172234] McLachlan K McNeil A Pei J , et al. (2019) Prevalence and characteristics of adults with fetal alcohol spectrum disorder in corrections: a Canadian case ascertainment study. BMC Public Health 19(1): 43. 30626356 10.1186/s12889-018-6292-x PMC6325737

[bibr18-17446295231172234] Ministry of Health (2022) Fetal alcohol spectrum disorder. Available at: https://www.health.govt.nz/our-work/diseases-and-conditions/fetal-alcohol-spectrum-disorder (accessed 5 April).

[bibr19-17446295231172234] Moore EM Riley EP (2015) What Happens When Children with Fetal Alcohol Spectrum Disorders Become Adults? Curr Dev Disord Rep 2(3): 219–227. 26543794 10.1007/s40474-015-0053-7 PMC4629517

[bibr20-17446295231172234] Mukherjee R Wray E Commers M , et al. (2013) The impact of raising a child with FASD upon carers: findings from a mixed methodology study in the UK. Adoption & Fostering 37(1): 43–56.

[bibr21-17446295231172234] Mukherjee R Wray E Curfs L , et al. (2015) Knowledge and opinions of professional groups concerning FASD in the UK. Adoption & Fostering 39(3): 212–224.

[bibr22-17446295231172234] Mutch R Watkins R Jones H , et al. (2013) Fetal Alcohol Spectrum Disorder: Knowledge, attitudes and practice within the Western Australian justice system. *Perth, Australia: Telethon Institute for Child Health Research* .

[bibr23-17446295231172234] Passmore HM Mutch RC Watkins R , et al. (2021) Reframe the Behaviour: Evaluation of a training intervention to increase capacity in managing detained youth with fetal alcohol spectrum disorder and neurodevelopmental impairments. Psychiatry, Psychology and Law 28(3): 382–407. 10.1080/13218719.2020.1780643 PMC9067986 35530127

[bibr24-17446295231172234] Payne JM France KE Henley N , et al. (2011) Paediatricians' knowledge, attitudes and practice following provision of educational resources about prevention of prenatal alcohol exposure and Fetal Alcohol Spectrum Disorder. Journal of Paediatrics and Child Health 47(10): 704–710. 21449899 10.1111/j.1440-1754.2011.02037.x

[bibr25-17446295231172234] Petrenko CLM Tahir N Mahoney EC , et al. (2014) Prevention of secondary conditions in fetal alcohol spectrum disorders: identification of systems-level barriers. Maternal and child health journal 18(6): 1496–1505. 24178158 10.1007/s10995-013-1390-y PMC4007413

[bibr26-17446295231172234] Popova S Lange S Shield H , et al. (2019) Prevalence of fetal alcohol spectrum disorder among special subpopulations: a systematic review and meta-analysis. Addiction 114: 1150–1172. 30831001 10.1111/add.14598 PMC6593791

[bibr27-17446295231172234] Reid N Kippin N Passmore H , et al. (2020) Fetal alcohol spectrum disorder: the importance of assessment, diagnosis and support in the Australian justice context. Psychiatry, Psychology and Law 27: 265–274. 10.1080/13218719.2020.1719375 PMC7476625 32944126

[bibr28-17446295231172234] Roozen S Stutterheim SE Bos AER , et al. (2022) Understanding the Social Stigma of Fetal Alcohol Spectrum Disorders: From Theory to Interventions. Foundations of Science 27(2): 753–771.

[bibr29-17446295231172234] Streissguth AP Bookstein FL Barr HM , et al. (2004) Risk factors for adverse life outcomes in fetal alcohol syndrome and fetal alcohol effects. Journal of Developmental & Behavioral Pediatrics 25(4): 228–238. 15308923 10.1097/00004703-200408000-00002

[bibr30-17446295231172234] Terry G Hayfield N (2020) Reflexive thematic analysis. In: Ward MRM Delamont S (eds) Handbook of Qualitative Research in Education. 2nd Edition ed. Cheltenham, UK: Edward Elgar Publishing.

[bibr31-17446295231172234] Wolff KR (1998) Social workers' knowledge about fetal alcohol syndrome, fetal alcohol effect, and alcohol-related birth defects. California State University, Long Beach.

[bibr32-17446295231172234] Wouldes T (2009) What health professionals know and do about alcohol and other drug use during pregnancy. Alcohol Healthwatch. 1–94.

